# Free vascularized fibula transfer for total wrist arthrodesis: A solution for failed wrist arthrodesis after wrist hemiarthroplasty extraction

**DOI:** 10.1016/j.jpra.2025.08.016

**Published:** 2025-08-21

**Authors:** Daniel Reiser, Mattias Hedspång, Marcus Sagerfors

**Affiliations:** Department of Orthopedics and Hand Surgery, Faculty of Medicine and Health, Örebro University, Södra Grev Rosengatan, 701 85 Örebro, Sweden

**Keywords:** Arthrodesis, Arthroplasty, Free fibula transfer, Wrist

## Abstract

Salvage radiocarpal arthrodesis is an option in the treatment of failed wrist arthroplasty. If bony union is achieved the results can be comparable to primary wrist arthrodesis. Substantial bone loss and decreased vascularity of the bone may be an issue in some cases. The present case report describes the case of a 58-year-old man with posttraumatic wrist arthritis after an intraarticular distal radius fracture. He was treated with a wrist hemiarthroplasty which ultimately failed and was converted to a radiocarpal arthrodesis. After two failed arthrodeses with bone grafting and a dorsal locking plate, we performed a free vascularized fibula transfer which resulted in bony union and a pain-free wrist. Free vascularized fibula transfer may be a salvage option in select cases of failed radiocarpal arthrodesis.

## Introduction

Vascularized bone grafts have become integral to bone reconstruction strategies in hand surgery and orthopedics, and different indications are well documented in the literature.^1^ Various application areas have evolved as the understanding of the biology of living bone transfer has improved. Two main indications have been described using a free vascularized fibula transfer (FVFT) in wrist reconstructive surgery: wrist reconstruction in adults after giant cell tumor, and vascularized proximal fibular epiphyseal transfer (VFET) for reconstruction of the distal radius in children.

Surgical treatment options in failed total wrist arthroplasty (TWA) include salvage by implant revision, or conversion to a radio-carpal arthrodesis (RCA). The outcome after converting a failed TWA to an RCA can improve wrist force and decrease pain; if bony union is achieved, the clinical outcome is comparable to the outcome after a primary RCA.^2^ A recent paper noted a high frequency of complications after wrist arthrodesis and noted better results for primary arthrodesis compared to arthrodesis after failed TWA.^3^ Some findings indicate that RCAs after TWAs can involve technical difficulties, such as substantial bone loss following implant removal and poor bone quality in rheumatic patients.^4^ Studies using an FVFT for radiocarpal arthrodesis (RCA) of the wrist are sparse and focused mainly on post-infectious conditions.^5^

We present a case of a failed wrist hemiarthroplasty converted to an RCA without bony union. After two unsuccessful attempts at an RCA with bone grafting and a dorsal locking plate, a free vascularized fibula transfer was successfully performed to achieve bony union.

## Case presentation

**Figure 1:**
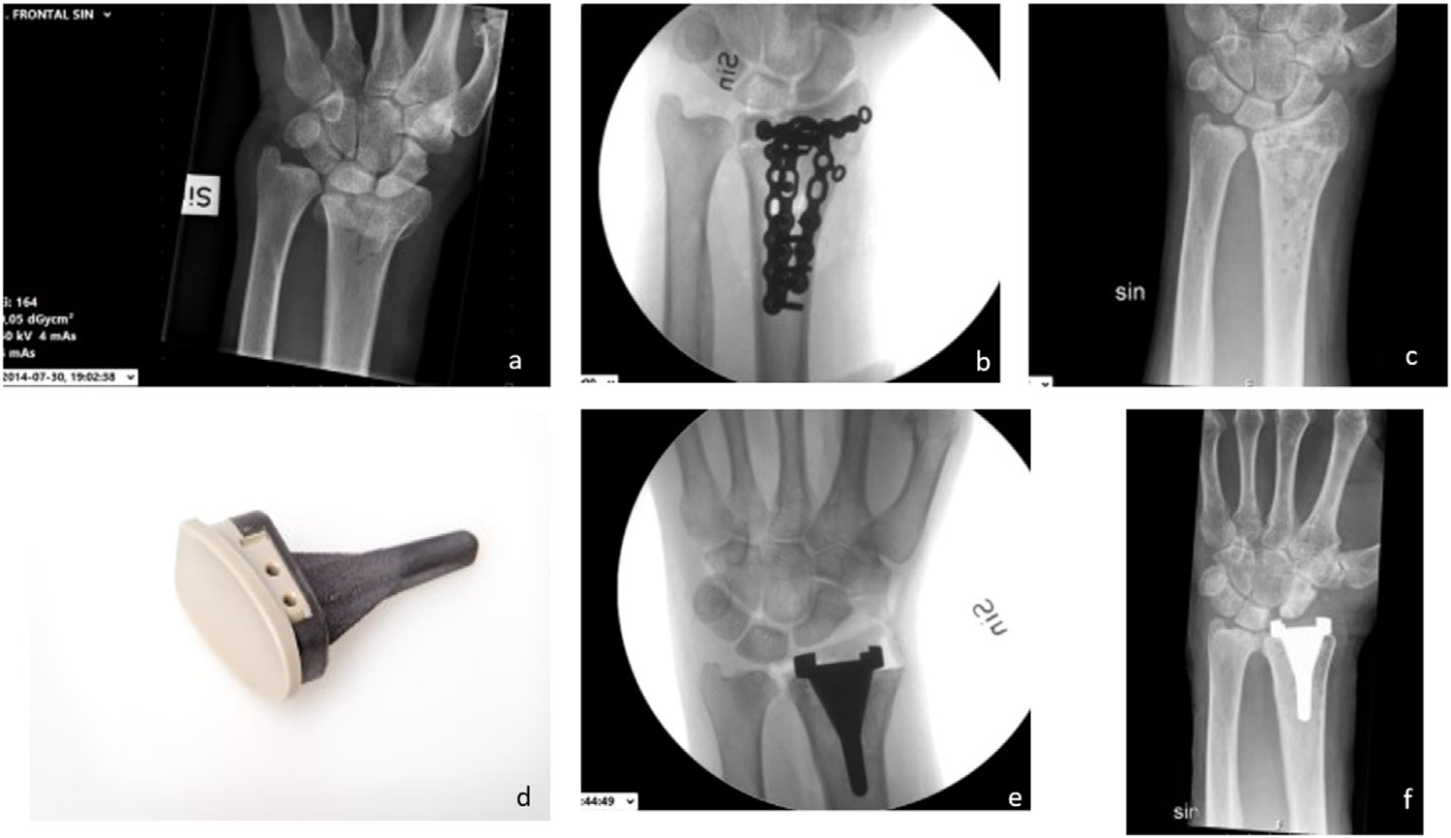
Initial radiographs: a) Distal radius fracture; b) fracture fixation with combined plating; c) after hardware removal; d) the new hemiarthroplasty design; e) perioperative radiographs after insertion of the hemiarthroplasty; f) radiographs one year postoperatively showing ulnar drift of the carpus and carpal impingement on the implant.

We present the case of an otherwise healthy worker who fell three meters from a truck at the age of 58, sustaining an AO (Arbeitsgemeinschaft für Osteosynthesefragen) type C3 distal radius fracture of the dominant left wrist. He underwent acute surgery with combined plating and grafting with porous beta-tricalcium phosphate bone substitute (Vitoss™ Stryker, Portage, MI, USA) due to a major metaphyseal bone defect of the distal radius. Following issues with adhesions and synovitis, hardware removal was performed eight months later. The patient subsequently developed posttraumatic radiocarpal osteoarthritis. Two years after the initial trauma, it was decided to provide him with a newly developed wrist hemiarthroplasty replacing the chondral surface of the radius. The implant is not commercially available and uses a central core of stainless steel, a porous titanium coating, and a thin outer layer of tantalum (Figure 1). The articulation is made of carbon-fiber–reinforced poly-ether-ether-ketone (CFR-PEEK). The transplant was tested in a pilot study with 20 cases. Mid-term follow-up for this hemiarthroplasty is available.^6^

**Figure 2:**
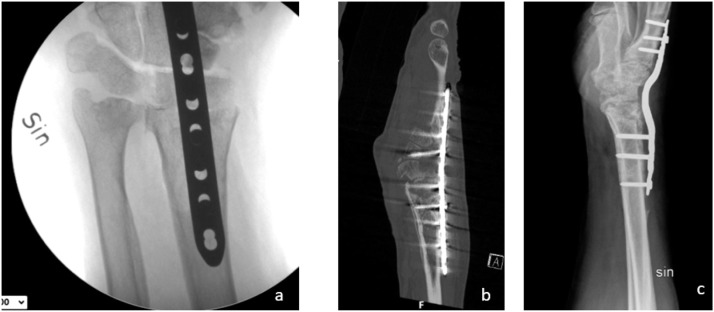
Radiographs after first and second radiocarpal arthrodeses: a) perioperative radiographs after radiocarpal arthrodesis; b) no bony union one year after the first procedure; c) no bony union one year after the second procedure.

The hemiarthroplasty did not provide a pain-free wrist and the patient was unable to work. The patient underwent three further procedures after the hemiprosthesis procedure: two tenolysis/neurolysis and removal of the lunate. The lunate bone was removed due to the onset of ulnar drift of the hemiprosthesis. Due to persistent wrist pain, a decision was made to remove the hemiarthroplasty and conduct an total wrist arthrodesis. During the removal, wear on the CFR-PEEK component was noted. The RCA was performed four years after the trauma using a dorsal locking plate (DePuy Synthes, Raynham, MA, USA) and corticocancellous allograft. There was no bony union. Two years later, another RCA attempt was made using cancellous bone from the iliac crest and a locking plate. Numerous tissue cultures were taken but all were negative for bacteria. No bony union was seen in follow-up CT scans (Figure 2). The hardware was removed. After a thorough discussion with the patient, he was offered an FVFT to provide healthy, well-vascularized bone to the wrist to promote bony union. Oral and written informed consent was obtained from the patient.

### Surgical method

In preparation for the operation, CT angiography of the left forearm and the right lower leg was performed. In our experience, the contralateral lower leg is preferred as a donor site from a postoperative, physiotherapeutic point of view.

**Figure 3:**
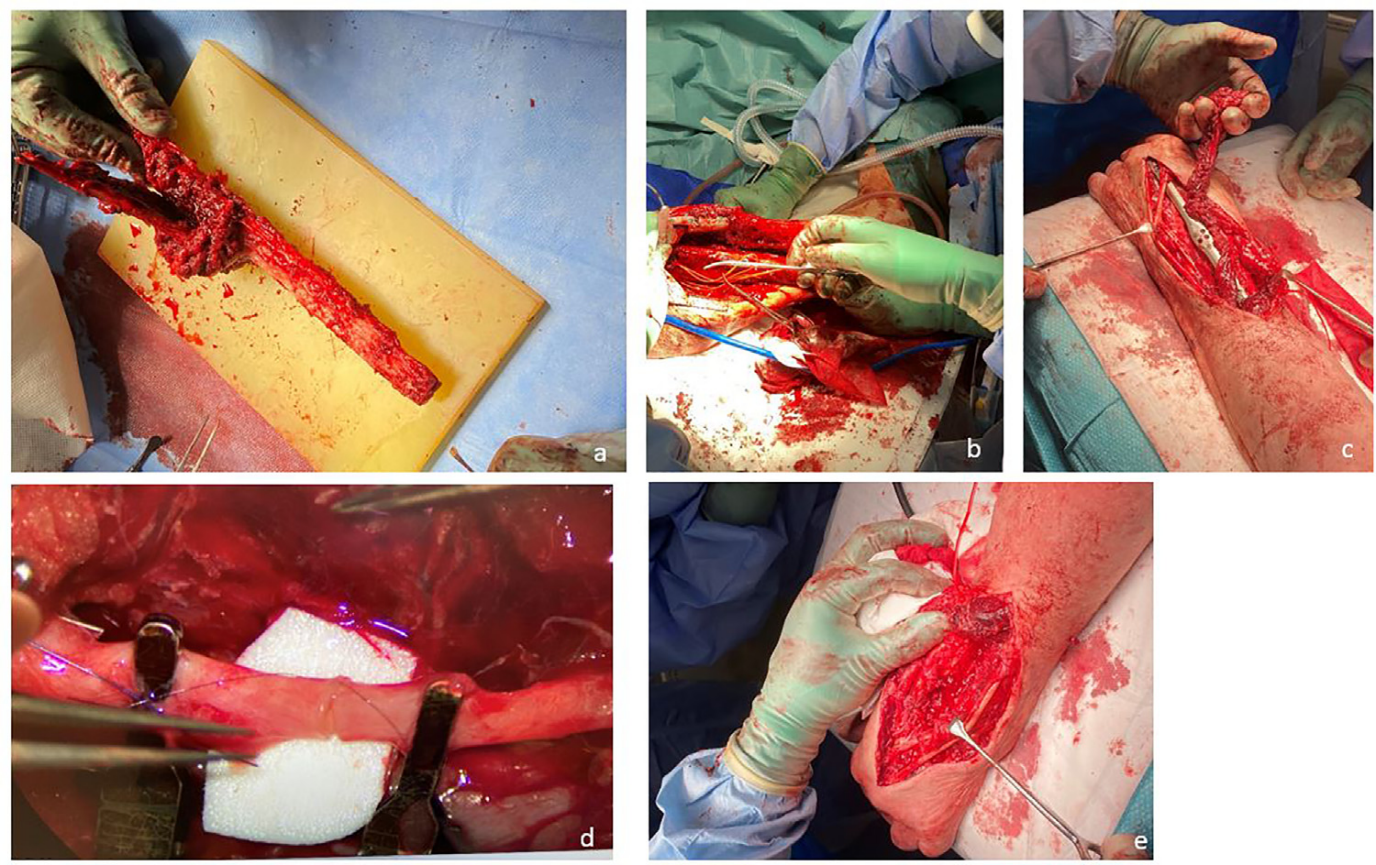
a) CT angiography underarm; b) CT angiography lower leg.

**Figure 4:**
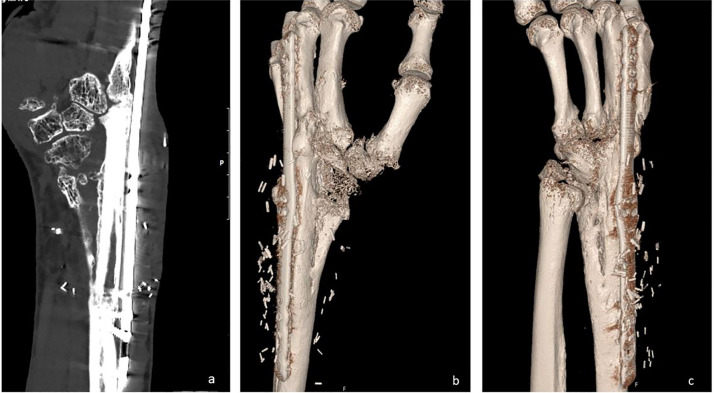
Free vascularized fibula transfer and fixation with a wrist spanning plate: a) Fibulatransfer; b) Right fibula to left radius; c) Osteosynthesis with a spanning plate; d) fibular vessels were sutured to the radial artery and cephalic vein; e) after flap-suture before wound closing with meshed split-skin graft.

The fibula was harvested with the vascular pedicle under Doppler control. The radius was opened about 7 cm dorsally for fenestration, and the non-union site was thoroughly debrided to expose healthy bleeding bone of the radius and third metacarpal (Figure 3). The fibula was fitted to the floor of the third metacarpal bone and embedded in the radius recess. Bony fixation was done using a dorsal wrist-spanning locking plate (Acu-Loc™ wrist-spanning plate; Swemac Inc., Linköping, Sweden) fixated in the proximal radius and the third metacarpal. The fibular vessels were sutured end to side to the radial artery and cephalic vein under the microscope using 8.0 nylon sutures and venous couplers. The wound edges were adapted using regular 4.0 Ethilon and the remaining wound was covered with a meshed split-skin graft to keep the external pressure on the pedicle as low as possible. Postoperatively, the wrist was immobilized in a volar splint. CT was performed after 2, 4, and 15 months to assess bony union. The CT scan after four months showed initiation of bony union of the fibula to the radius and to the third metacarpal. At the latest follow-up CT scan 15 months postoperatively, the patient was able to carry out his manual work again without pain (Figure 4). He rides a motorcycle in his free time and can walk up to three kilometers without pain or any hindrance. Four months postoperatively, the quickDASH (Quick Disabilities of the Arm, Shoulder and Hand) score was 27 and the PRWE (patient rated wrist evaluation) score was 28, indicating substantial improvement compared with preoperative values. VAS during activity and at rest was 0. The range of motion was similar in the left and right feet and ankles, as were pronation and supination in the left and right forearms. Hand grip strength, assessed using a Jamar Hand Dynamometer (Biometrics Ltd., Newport, UK), differed considerably between the right and left sides but remained largely unchanged (Table 1).

**Table 1:** Functional outcome at 4 months post-operatively.

VAS at rest	0
VAS during activity	0
Pronation	0/70
Supination	0/70
Jamar hand grip strength dx	60,6 kg
Jamar hand grip strength sin	14,5
PRWE score	28
Quick-DASH score	27
Balance test on one leg in 30 s	
Right	9 stop
Left	0 stop

## Discussion

We present an unusual case of a patient with posttraumatic osteoarthritis after a distal radius fracture in whom joint replacement with a wrist hemiarthroplasty failed, as did subsequent attempts at an RCA. Bony union of the RCA could finally be achieved using a free fibula transfer. TWA is a motion-preserving alternative to RCA for wrist osteoarthritis. Previous TWA designs have demonstrated high revision rates, often involving the carpal component. Improved implant survival rates and better patient-reported outcomes have been reported with modern designs, but complications after TWA requiring additional surgery still remains an issue.^7^ A recent randomized controlled trial comparing two modern TWA designs found equally improved function with both implants, but nearly a third of patients underwent additional surgery due to complications.^8^ A hemiarthroplasty, replacing only the radial articulation, could potentially avoid some of the issues related to carpal component loosening. The mid-term follow-up using the new hemiarthroplasty is available, and revisions and ulnar drift have been seen in this cohort, as was also noted in this patient.^6^

We have no explanation for the lack of healing of the RCA. The patient was non-smoker, otherwise healthy, and showed high compliance. We found that after hemiprosthesis removal, the distal end of the cortical radius had very limited viability, which may have been due to the prosthesis design.

Conversion of TWAs to RCAs has been described in various studies, but can be more challenging than primary RCAs, often due to substantial bone loss and osteolysis.^3^^,^^4^ In this case, the situation was assessed and the reason for the failed bony union was likely multifactorial. The initial fixation of the patient’s distal radius fracture was done using combined plating, which entails substantially more periosteal stripping of the radius than does regular volar plating.^9^ The metaphyseal bone defect was filled with beta-tricalcium phosphate bone substitute. Although some clinical data indicate a good effect in combination with synthetic bone grafting, it cannot be excluded that the bone substitute could have had a negative impact in this case as no bone grafting was done at the initial fracture fixation. In addition, the hemiarthroplasty had an articulating liner of CFR-PEEK.^6^ A recent study on the Motec TWA found that the metal vs CFR-PEEK articulation generated some material loss in vivo.^10^ The hemiarthroplasty design in this study has bone articulating against CFR-PEEK so the articulations are not directly comparable. The ulnar drift seen on postoperative radiographs, with the lunate impinging on the ulnar edge of the CFR-PEEK liner, could potentially generate PEEK particles, whose impact on the bony biology is largely unknown.

## Conclusion

Using an FVFT in combination with a wrist-spanning plate helped achieve bony union in this case. The patient has a pain-free, stable wrist with no symptoms from the lower leg/ankle after the procedure. We have no clear explanation for the lack of healing in the RCA. The patient was a non-smoker, otherwise healthy, and demonstrated high compliance throughout treatment. Following the removal of the hemiprosthesis, we observed that the distal end of the radial cortex showed very limited viability, which may have been related to the design of the prosthesis. If poor bone viability was indeed the underlying cause, this technique may have a role in selected cases where similar conditions are present. This case demonstrates that an FVFT can be a solution to achieve bony union of the wrist in select cases (Picture 5).

**Picture 5:**
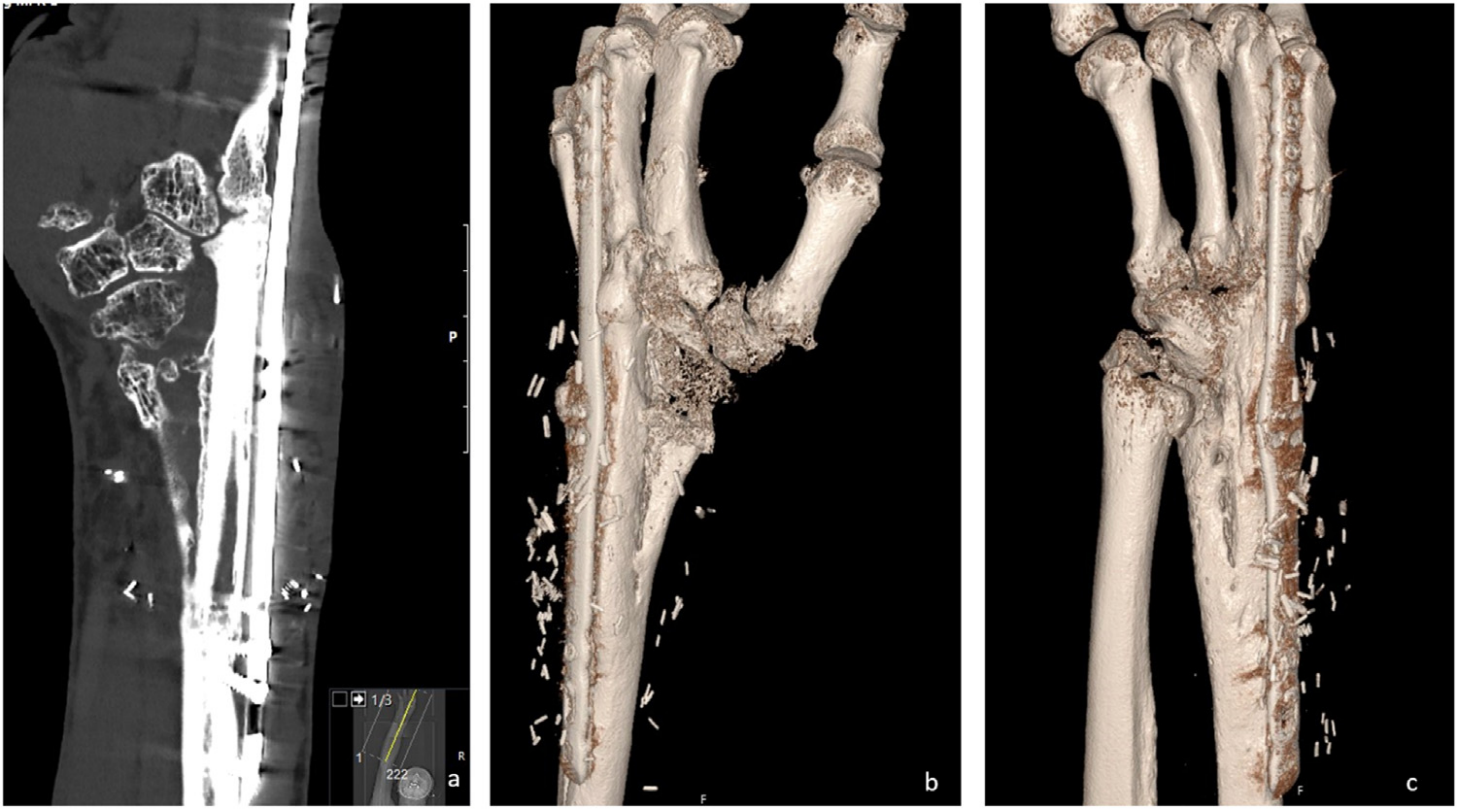
after Fibula transfer a: CT 12 month after the procedure b and c: 3D CT 12 month after the procedure.

## Ethical approval

The study was conducted in accordance with the Declaration of Helsinki (as revised in 2013). Oral and written informed consent has been given by the patient. Radiographs and photos are anonymized.

## Declaration of competing interest

The authors report there are no competing interests to declare.
